# Eliminating the monitoring period with subcutaneous daratumumab: a single-center experience

**DOI:** 10.1038/s41408-023-00801-1

**Published:** 2023-02-20

**Authors:** Kathryn T. Maples, Kevin H. Hall, Nisha S. Joseph, Craig C. Hofmeister, Vikas Gupta, Madhav V. Dhodapkar, Jonathan L. Kaufman, Ajay K. Nooka, Sagar Lonial

**Affiliations:** Department of Hematology and Medical Oncology, Winship Cancer Institute, Emory University School of Medicine, Atlanta, GA USA

**Keywords:** Myeloma, Myeloma

Dear Editor,

Daratumumab is a first-in-class human IgG1 kappa CD38-targeted monoclonal antibody that has become a critical component in the treatment of newly diagnosed multiple myeloma (NDMM), relapsed and refractory multiple myeloma (RRMM), and AL Amyloidosis. Anti-CD38 antibodies induce cell death through multiple mechanisms including: antibody-dependent cell-mediated cytotoxicity (ADCC), antibody-dependent cellular phagocytosis, complement-dependent cytotoxicity, induction of apoptosis, and modulation of CD38 enzyme activity [1]. In recent years, we have also seen daratumumab be utilized to treat refractory post-transplant autoimmune hemolytic anemia, POEMS disease, and relapsed or refractory Natural killer/T-cell lymphoma (NKTCL) [2, 3].

Daratumumab is administered as an intravenous (IV) infusion at a weight based dose of 16 mg/kg, and a historical challenge to treatment with daratumumab is the high incidence of infusion-related reactions (IRRs) [4]. Smooth muscle cells in the airway express CD38 and IRR secondary to daratumumab often manifest with pulmonary complications including cough, dyspnea, throat irritation, and congestion [5]. In the pivotal phase 2 SIRIUS trial, which led to the FDA approval of daratumumab as monotherapy for RRMM, IRR occurred in 42% of patients (5% being grade 3 and 0% grade 4) [6]. Due to the risk for IRRs, the first dose of IV daratumumab is administered very slowly and can take on average 7.6 h to complete [4]. Once tolerated, subsequent infusions of daratumumab can be administered via the rapid infusion protocol, which is still a minimum of 90 min [7]. In May of 2020, daratumumab and hyaluronidase-fihj was FDA approved, which offers a novel subcutaneous (SC) administration for daratumumab. In the phase 3 COLUMBA trial, SC daratumumab yielded a similar overall response rate (41% vs. 37%) and fewer infusion related reactions (13% vs. 34%) compared to IV daratumumab, respectively [8]. SC daratumumab offers shorter preparation and administration time with less risk for reactions, which can improve the patient experience and has become the preferred daratumumab formulation at the Winship Cancer Institute.

The prescribing information for SC daratumumab recommends that patients be monitored after cycle 1 day 1; however, it does not specify a recommended length of time [9]. When SC daratumumab was originally introduced at Winship, we implemented a 3.5 h observation time for cycle 1 day 1 only based on the COLUMBA trial [8]. With additional data from recent trials, the pooled rate of IRR with SC daratumumab is reported to be <10%, which brings the 3.5 h observation time into question. Additionally, Davis et al. previously reported on their single center experience of safely reducing the observation period to 2 h after cycle 1 day 1, and proceeding without observation for subsequent doses [10].

This single-center, retrospective analysis, approved by the Institutional Review Board (IRB) at Emory University, sought to find the optimal observation time (if any) after the administration of SC daratumumab in order to balance safety of the drug with convenience for the patient and increase infusion chair availability. This study occurred in a two-step process from May 2022 through September 2022: Step 1. The observation time was reduced from 3.5 h to 2 h after cycle 1 day 1 only. After implementing the 2-hour observation time for an 8-week period, we evaluated the frequency of reactions for all patients who received day 1 daratumumab as well as any phone calls to the center within 24 h. Step 2. Given the IRR rate from step 1 was <10%, we then reduced to a no observation time and repeated the process. A patient education handout was developed to inform patients on the signs and symptoms of an IRR, including instructions on when to call and who to call if these symptoms were to occur. This handout was given to all patients in the 0-hour infusion group in the infusion center on the day of their first SC daratumumab injection. Our institutional pre-medication protocol included acetaminophen 650 mg, diphenhydramine 50 mg, montelukast 10 mg, and dexamethasone 20–40 mg administered orally 30 min prior to the first injection. Acetaminophen and diphenhydramine are also given prior to the second injection along with a corticosteroid if part of the treatment regimen, but no pre-medications are given starting with the third injection outside of the therapeutic corticosteroid except in those with a previous reaction. No post-medications were given to the patients included in this analysis, which is in alignment with our institution standard. Additionally, for patients on a combination regimen, daratumumab was the last medication to be administered on the treatment day. The rationale for this decision was to avoid additional medications being administered in the 2-hour observation period, which could have confounded any reactions; therefore, those in the 0-hour observation group were discharged immediately following the daratumumab injection and those in the 2-hour observation group were discharged following the completion of the monitoring window.

A total of 64 daratumumab naïve patients were included during the 16-week study period. Twenty-nine (45%) and 35 (55%) patients were observed for 2 h and no observation (0-hour) after the first injection, respectively. Thirty-two (50%) patients received SC daratumumab for NDMM, 23 (36%) patients were treated for RRMM, and 6 (9%) had AL Amyloidosis. Fifty-nine percent were female, the median age was 64 years (range 34–86), and median weight was 79 kg (range 46–194). Patient characteristics are summarized in Table 1.

**Table 1:** Patient Characteristics.

	2-hour Observation (*n* = 29)	0-hour Observation (*n* = 35)
Age, median (range)	61 years (34–86)	67 years (44–86)
<65 years	19	15
≥65 years	10	20
Sex		
Female	19	19
Male	10	16
Race		
African American	8	14
Caucasian	18	14
Asian	1	3
Unknown	2	4
Weight, median (range)	74 kg (46–164)	84 kg (50–194)
<65 kg	7	8
65–85 kg	12	10
>85 kg	10	17
Indication		
Newly diagnosed MM	18	14
Relapsed/refractory MM	6	17
AL Amyloidosis	2	4
Other (lymphoma, POEMS)	3	0
Comorbidities		
Cardiac (CAD, CHF, Afib)	8	9
COPD/Asthma	0	2
HTN	11	19
Renal dysfunction		
CrCl >60 mL/min	18	26
CrCl 30–60 mL/min	7	6
CrCl <30 mL/min (Not on HD)	2	3
HD	2	0

*Afib* atrial fibrillation, *CAD* coronary artery disease, *CHF* congestive heart failure, *CrCl* creatinine clearance, *COPD* chronic obstructive pulmonary disease, *HD* hemodialysis, *HTN* hypertension, *MM* multiple myeloma, *POEMS* Polyneuropathy, organomegaly, endocrinopathy, monoclonal-protein, skin changes.

Three patients (4.7%) had an IRR, all of which were grade 1 or 2 and occurred in 2 patients in the 2-hour observation group and 1 patient in the 0-hour observation group. Due to the fixed dose with SC daratumumab, it is worth noting that the 2 patients who experienced a reaction in the 2-hour observation group were in the 65–85 kg weight range group. The 1 patient with a reaction in the 0-hour group was <65 kg; however, this reaction was pain at the injection site, which is not a true IRR. All reactions were reversible with supportive care medications including famotidine, acetaminophen, diphenhydramine, and/or hydrocortisone. The most common IRRs were rash and chills. No serious treatment-related IRRs (grade ≥3) or IRRs leading to discontinuation of daratumumab occurred. Additionally, 1 patient in the 2-hour group had their reaction once home after the 2-hour observation and this was able to be managed with instruction over the phone via our nursing triage system. The previously mentioned education handout advises patients on when to call and when to seek immediate medical attention at the closest emergency room. Zero patients required an emergency room visit or hospitalization. An overview of the IRR details is presented in Table 2.

**Table 2:** Infusion Related Reaction Details.

	2-hour Observation (*n* = 29)	0-hour Observation (*n* = 35)
Reactions		
Any Grade	2	1
Grade 1-2	2	1
Grade 3-4	0	0
Reaction Type		
Rash	1	0
Chills	1	0
Pyrexia	0	0
Pain at injection site	0	1
Dyspnea	0	0
Treatment-discontinuation	0	0
Use of rescue meds		
In infusion	1	0
At home within 24 h	1	0
ER Visit	0	0
Hospitalization	0	0

*ER* emergency room.

With no difference observed in terms of efficacy, SC daratumumab has many advantages over the IV formulation including lower rates of IRRs and significantly shorter administration time [8]. Although the administration time is shorter, the prescribing information still recommends an observation period, which limits the full amount of time saved with the SC administration [9]. Due to the low rates of IRRs reported with SC daratumumab, we opted to eliminate our observation period via a step-wise approach, and found an all-grade IRR rate of 4.7% (0% grade 3 or higher). Reducing to a no-observation chair time with SC daratumumab can lead to cost savings, decreased time at the cancer center for the patient, increase in chair availability, and decreased burden on healthcare staff, all of which have proven to be critical during the COVID-19 pandemic and nursing shortages.

Lastly, when SC daratumumab was originally approved in May 2020 and patients at the Winship Cancer Institute were transitioned from IV to SC daratumumab, a five question patient survey was administered to patients who had received at least one dose of both IV and SC daratumumab and had consented under IRB approved protocol. Twenty-two patients completed the survey, which showed 73% strongly preferred SC, 14% preferred SC, 9% had no preference, and 4% strongly preferred IV. The top reasons for the preference included the treatment administration time and fewer administration related reactions.

The limitations of this study include its retrospective design and limited sample size given the short 16-week study period. Nevertheless, to our knowledge, this study is the first to assess a 0-hour observation period after first-dose SC daratumumab. We recommend that patients be properly counselled on the side effects and provided with guidance on when and who to call if any symptoms should occur so that they may be managed either at home with oral medications or sent to an emergency room for a severe reaction. We believe our data illustrate that patients can safely receive SC daratumumab with no observation period and patients prefer this administration route over IV.

## Data Availability

Data available on request from the authors.
